# Inter‐prefectural and urban–rural regional disparities in rectal cancer and rectal resections: A Japanese nationwide population‐based cohort study from 2014 to 2019

**DOI:** 10.1002/ags3.12865

**Published:** 2024-10-01

**Authors:** Masamitsu Kido, Tomohiro Arita, Katsutoshi Shoda, Hiroki Shimizu, Jun Kiuchi, Kenji Nanishi, Luying Yan, Eigo Otsuji

**Affiliations:** ^1^ Department of Orthopedic Surgery Inage Hospital Chiba Japan; ^2^ Division of Digestive Surgery, Department of Surgery, Graduate School of Medical Science Kyoto Prefectural University of Medicine Kyoto Japan; ^3^ First Department of Surgery, Faculty of Medicine University of Yamanashi Chuo Yamanashi Japan; ^4^ Department of Anesthesiology Brigham and Women's Hospital Boston Massachusetts USA

**Keywords:** database, epidemiology, rectal cancer, rectal resection, surgeon

## Abstract

**Aim:**

This observational study aimed to elucidate the regional disparities in rectal cancer (RC) and rectal resections (RRs) across Japan.

**Methods:**

The annual incidence of RC, and number of all RRs and board‐certified surgeons by the Japan Society for Endoscopic Surgery were examined by prefecture in Japan from 2014 to 2019. The surgical approaches were broken down by open and laparoscopic. Disparities in 47 prefectures and urban–rural disparities were evaluated using the Gini coefficient and unpaired *t*‐test.

**Results:**

The annual national average incidence of RC was 50 127 and the number of all RRs was 39 903. Gini coefficients for RC, and laparoscopic and all RRs were <0.2, indicating low inequality. There was no significant difference between urban and rural prefectures in the number of RRs, despite a significantly higher incidence of RC in rural prefectures and a significantly higher number of board‐certified surgeons in urban prefectures (*p* < 0.05).

**Conclusion:**

RC and laparoscopic and all RRs exhibited minimal inter‐prefectural disparities. The urban–rural analysis revealed significant differences in the incidence/number of RC and board‐certified surgeons between urban and rural prefectures, despite minor differences in RRs regardless of approach. This pattern suggests a potential migration of surgical services from rural to urban areas. This preliminary study is expected to contribute to a basic epidemiological database for RC and RRs.

## Graphical abstract

This study aimed to clarify regional disparities in rectal cancer and rectal resections, using a Japanese nationwide database. There was no significant difference between urban and rural prefectures in the number of rectal resections, despite a significantly higher incidence of rectal cancer in rural prefectures and a significantly higher number of board‐certified surgeons in urban prefectures. This pattern suggests a potential migration of surgical services from rural to urban areas.

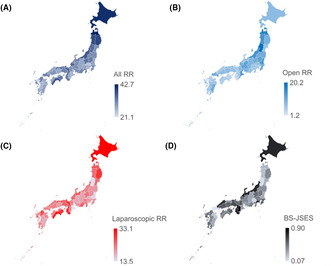

## 1 INTRODUCTION

The incidence of rectal cancer (RC) has been increasing in developing countries, while declining or plateauing in developed countries, likely reflecting improvements in screening.^1^ In 2020, Japan reported the second‐highest total incidence of cases (50 668) after China (244 550), and the third highest incidence per 100 000 population (40.1) after Portugal (48.6) and Latvia (41.4).^2^ According to the 2021 cancer death statistics in Japan,^3^ RC ranked seventh in mortality among both men and women, with a total of 15 645 deaths. The incidence and mortality rates have been increasing annually in Japan, likely indicative of the aging population.^4^

Epidemiological studies in Japan have demonstrated an increasing trend in the total number of colorectal surgeries, specifically low anterior resections, from 2011 to 2019.^5^ While numerous epidemiological reports about colorectal surgeries have been published worldwide, there is a paucity of research on localized regional disparities on a national level. In particular, there have been no epidemiological studies focusing on inter‐prefectural differences in Japan. Investigations have reported localized regional disparities in the distribution of physicians in most Organization for Economic Co‐operation and Development countries,^6^, ^7^, ^8^ often showing increased numbers in urban areas compared to rural ones. Considering Japan's rapidly aging population, it is imperative to gain a comprehensive understanding of such disparities in RC care.

The standard surgical management for RC involves total mesorectal excision,^1^, ^9^ resecting the entire mesorectum just above the anal canal^10^, ^11^. The utilization of laparoscopic surgery as an alternative to open surgery has been a topic of debate, with two randomized controlled trials supporting its efficacy,^12^, ^13^ while another two randomized controlled trials remained concerning, especially regarding total mesorectal excision and circumferential resection margin.^14^, ^15^

In Japan, the decision to select laparoscopic rectal resection (RR) is influenced by multiple factors, including tumor characteristics such as location and stage, patient‐specific factors like obesity and previous laparotomy history, and the surgeon's expertise and proficiency.^11^ In 2019, using the American College of Surgeons National Surgical Quality Improvement Program (ACS‐NSQIP) database, Davis et al raised concerns regarding the increasing use of laparoscopic resection for RC, despite its equivocal benefit.^16^ However, in Japan similar results of the increasing use of laparoscopic resection have been shown in the National Clinical Database,^5^ which was established in 2010 by several surgical societies in Japan, and a questionnaire survey report.^17^, ^18^

The purpose of this study was to investigate the contemporary patterns in RC and RRs across different prefectures in Japan. The study makes use of the National Database of Health Insurance Claims and Specific Health Checkups (NDB),^19^, ^20^, ^21^ which contains >95% of the country's data on medical insurance claims. Additionally, the study aimed to explore the availability of board‐certified surgeons by the Japan Society for Endoscopic Surgery (BS‐JSES) in each prefecture.

## 2 METHODS

### 2.1 Study design and data sampling technique

According to the Japanese ethical guidelines, this study did not require institutional board approval or informed consent because all data was obtained from publicly available data. To fully protect the anonymity of the patients, the NDB Open Data did not report data for procedures that occurred fewer than 10 times in a year.

We used three public open‐source databases: the Cancer Information Database,^22^ the NDB Open Data Japan,^23^ and the Ministry of Internal Affairs and Communications statistics (MIAC),^24^ to analyze the incidence of RC and the number of RR and BS‐JSES, in the 47 Japanese prefectures from 2014 to 2019. We chose to concentrate on trends before Coronavirus Disease 2019; hence, data from 2020 and beyond was excluded. MIAC demographic data^24^ was used to compute the rate per 100 000 people, and to quantify aging as the number of people aged ≥65 y.

### 2.2 Data sampling methods

We used the ICD‐10 codes C19‐C20 (malignant neoplasm of rectosigmoid junction and rectum) to identify RC cases. The number rate of BS‐JSES per 100 000 people was calculated from publicly available data on the JSES homepage (https://www.jses.or.jp/) and the MIAC data.^24^ The website lists the names, work locations, years of certifications, and subspecialities (esophagus, stomach, colon, etc.) of BS‐JSES by prefecture. We specifically targeted BS‐JSES with a subspecialty focus on the colon. The breakdown of RR was examined by site: high anterior resection, low anterior resection, very low anterior resection, resection with transanal anastomosis, and abdominoperipheral resection. We classified surgical approaches as open, laparoscopic, or all (encompassing both approaches). We included RR with the following surgical codes:

K740 Rectal resection and amputation

1. High anterior resection
2. Low anterior resection
3. Very low anterior resection
4. Resection with transanal anastomosis
5. Abdominoperipheral resection

K740‐2 Laparoscopic rectal resection and amputation

1. High anterior resection
2. Low anterior resection
3. Very low anterior resection
4. Resection with transanal anastomosis
5. Abdominoperipheral resection

We also investigated the total number of RRs (all sites and approaches) stratified by age from 2014 to 2019, using the NDB Open Data Japan.^23^

To assess inter‐prefectural regional disparities, we computed Gini coefficients^20^, ^21^, ^25^ to analyze differences in the incidence of RC and the number of RR and BS‐JSES between prefectures. Gini coefficients are traditionally used to measure income and wealth distribution, providing a standardized tool to gauge the degree of distribution inequality. The Gini coefficients were categorized as low (<0.2), moderate (≥0.2, <0.3), high (≥0.3, <0.4), or extreme (≥0.4) inequality, with 0 representing complete equality and 1 indicating complete inequality.

### 2.3 Definition of urban and rural prefectures

In accordance with previous studies,^20^, ^21^, ^26^ prefectures were classified as urban or rural based on the following definition: a densely populated group consisting of seven prefectures with a population density of more than 1000 persons per unit area (Saitama, Tokyo, Chiba, Kanagawa, Aichi, Osaka, and Fukuoka, accounting for approximately half of the total population in Japan) vs. a sparsely populated group consisting of the remaining 40 prefectures.

### 2.4 Statistical analyses

The Jonckheere‐Terpstra trend test was used to examine whether the number of open, laparoscopic, and all RRs changed over the 6‐year study period. Data comparisons between urban and rural groups were performed using the unpaired *t*‐test.

Statistical significance was set at a two‐sided *p*‐value of <0.05. All statistical analyses were performed using EZR (Saitama Medical Center, Jichi Medical University, Saitama, Japan),^27^ a graphical user interface for R (The R Foundation for Statistical Computing, Vienna, Austria).

## 3 RESULTS

### 3.1 Total number of RR (all sites and approaches) stratified by age during 2014–2019

**FIGURE 1:**
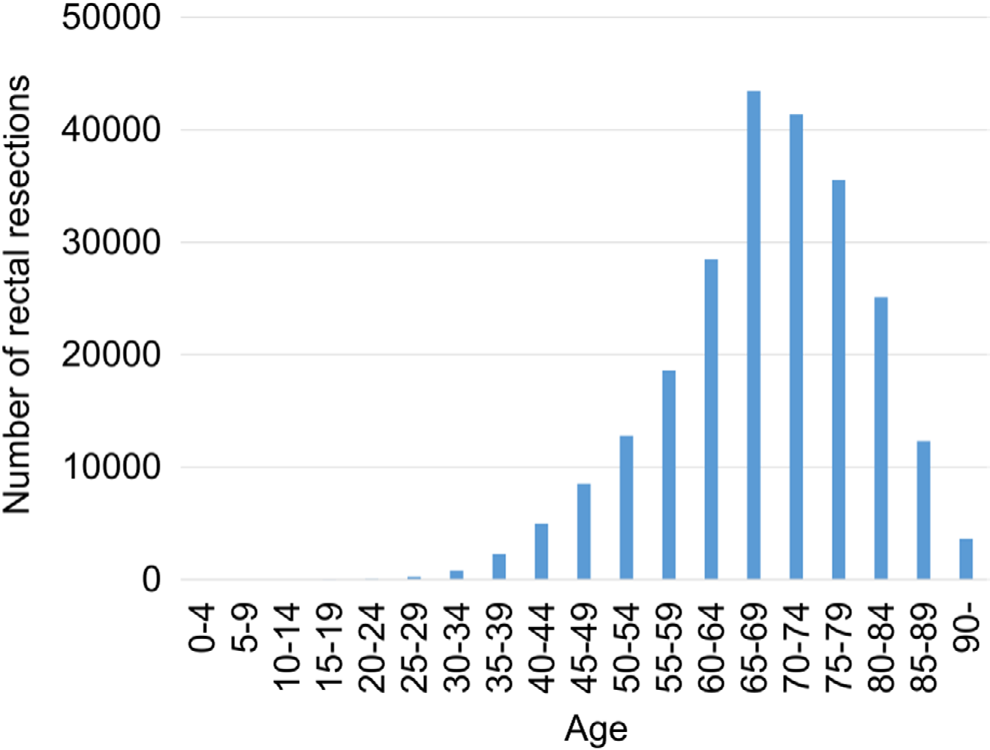
Total number of rectal resections (all sites and approaches) stratified by age during 2014–2019.

Regarding the age‐stratified number of RR (all sites and approaches) during 2014–2019, an unimodal distribution was observed, with a peak at 65–69 y (Figure 1). 67.8% of RR (all sites and approaches) occurred in people aged ≥65 y.

### 3.2 Trends in the annual number of RR during 2014–2019

**FIGURE 2:**
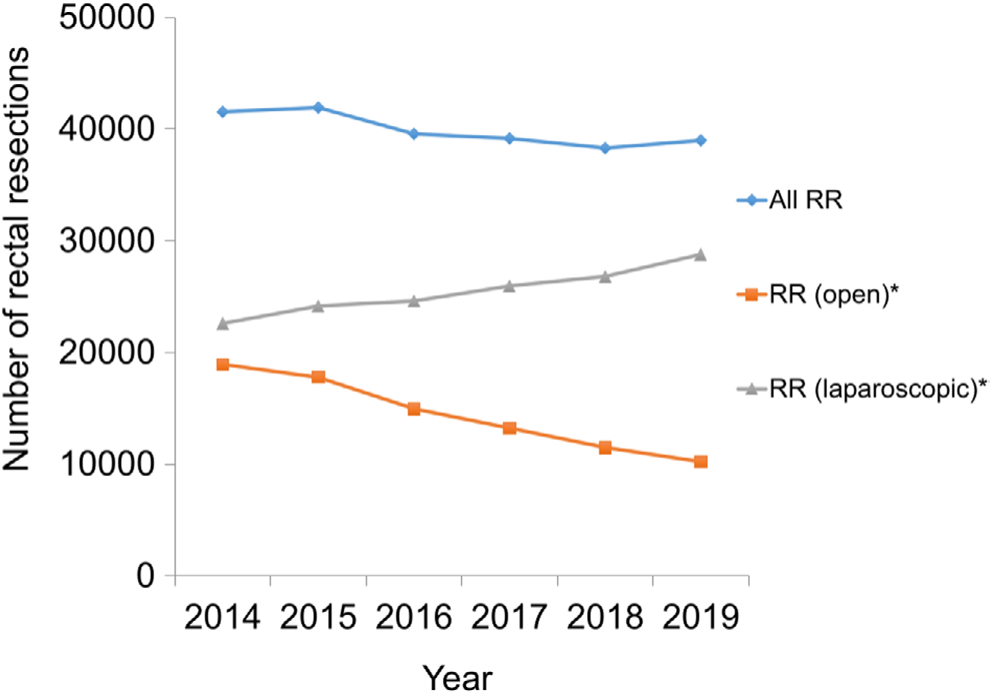
Trends in the annual number of rectal resections (open, laparoscopic, and all) in Japan from 2014 to 2019. RR, rectal resections. * A statistically significant trend (*p* < 0.05).

Over 6 years, the number of RR (laparoscopic) significantly increased (*p* < 0.01), whereas the number of RR (open) significantly decreased (*p* < 0.01) (Figure 2). No significant change was observed in the number of all RR (*p* = 0.06). The incidence of RC and the number of BS‐JSES remained approximately constant over the study period. Notably, the percentage of the number of laparoscopic RR of all RR was elevated from 54.4% (22 601/41 535, 2014) to 73.8% (28 781/38 979, 2019).

### 3.3 Basic statistics and Gini coefficients for RC, RR and BS‐JSES

**FIGURE 3:**
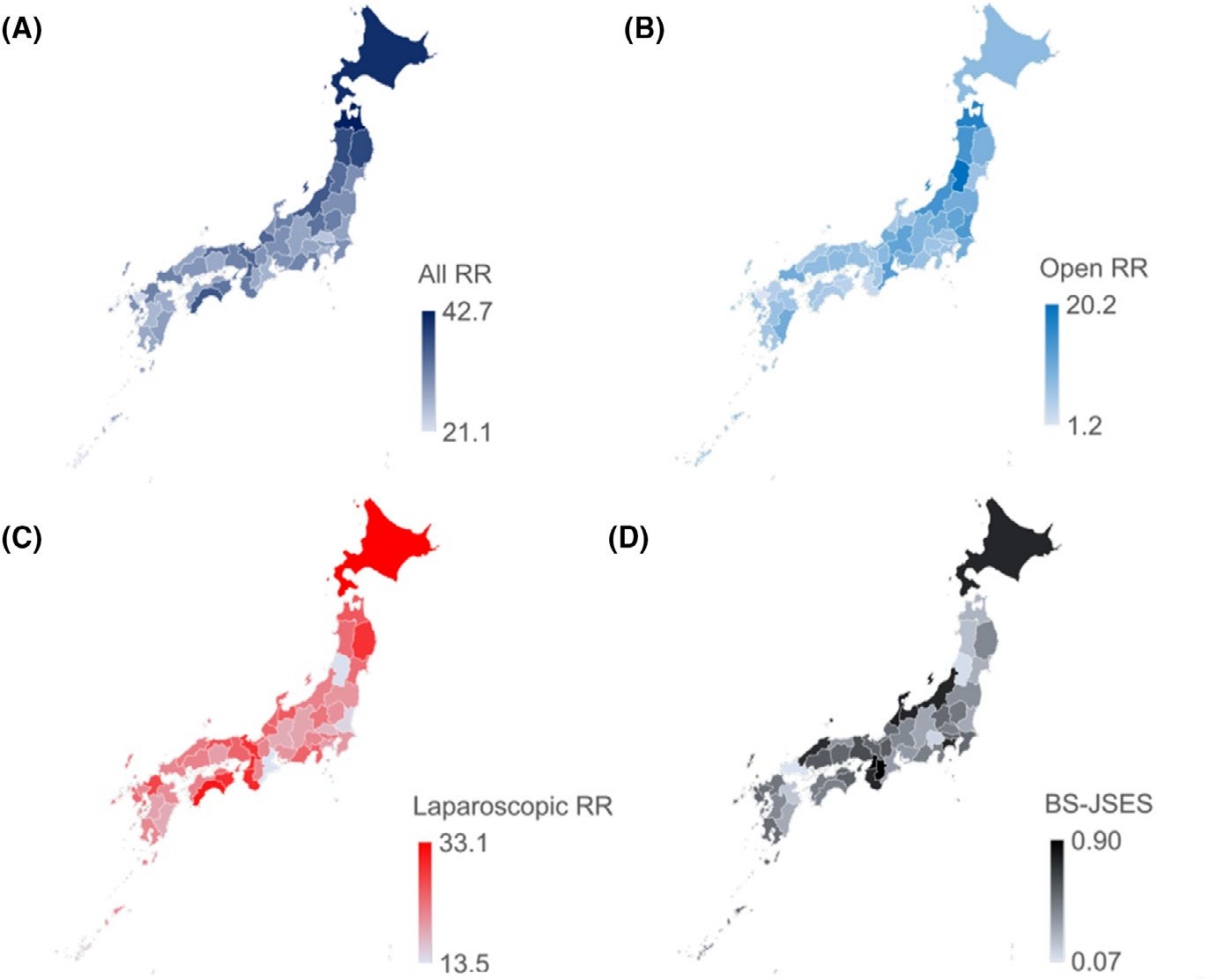
Heat maps for the number of (A) all rectal resections, (B) open rectal resections, (C) laparoscopic rectal resections, (D) board‐certified surgeons by the Japan Society for Endoscopic Surgery by prefecture in 2019. BS‐JSES, board‐certified surgeons by the Japan Society for Endoscopic Surgery; RR, rectal resections.

Table 1 shows the basic statistics and Gini coefficients for RC, RR, and BS‐JSES, using 47 prefectures as the unit of analysis. The annual national incidence/number of RC and all RR was 50 127 and 39 903, respectively. The heat maps show the latest 2019 data for the number of all RR, RR (open), RR (laparoscopic), and BS‐JSES per 100 000 person‐years (Figure 3A–D). The Gini coefficients were low (<0.2) for RC, all RR, laparoscopic RR, and BS‐JSES. However, the Gini coefficients for RR (open) displayed low‐moderate inequality, within the range of 0.16–0.23, accompanied by an upward trend in disparity.

**TABLE 1:** Basic statistics for the annual national incidence/number of the rectal cancer, rectal resections, and board‐certified surgeons by the Japan Society for Endoscopic Surgery across 47 prefectures.

	Annual national incidence/number	Incidence/number per 100 000 person‐years			Gini coefficient
		Average (95% CI)	Maximum	Minimum	
Rectal cancer					
2014	45 950	Not available	Not available	Not available	Not available
2015	47 054	Not available	Not available	Not available	Not available
2016	53 226	43.3 (41.8–44.9)	58.5	32.6	0.07
2017	51 241	41.6 (40.2–43.0)	56.1	32.8	0.06
2018	51 005	41.8 (40.3–43.3)	59.1	31.6	0.07
2019	52 287	42.9 (41.3–44.5)	59.1	31.4	0.07
All RR					
2014	41 535	27.7 (26.7–28.6)	37.5	19.2	0.06
2015	41 913	30.1 (29.1–31.2)	37.4	20.4	0.07
2016	39 555	31.3 (30.1–32.5)	42.5	20.8	0.07
2017	39 155	31.0 (29.8–32.2)	45.7	21.8	0.07
2018	38 284	30.3 (29.1–31.5)	40.9	20.7	0.07
2019	38 979	30.8 (29.6–32.1)	42.7	21.1	0.08
RR (open)					
2014	18 934	12.1 (11.0–13.2)	25.4	4.3	0.16
2015	17 756	11.6 (10.5–12.6)	23.5	6.0	0.16
2016	14 945	12.1 (11.0–13.2)	24.8	4.3	0.17
2017	13 208	10.7 (9.7–11.8)	22.2	4.5	0.18
2018	11 475	9.3 (8.3–10.3)	19.6	1.5	0.20
2019	10 198	8.3 (7.2–9.3)	20.2	1.2	0.23
RR (laparoscopic)					
2014	22 601	15.6 (14.7–16.5)	22.0	8.3	0.11
2015	24 157	18.6 (17.4–19.8)	27.4	9.5	0.12
2016	24 610	19.2 (18.1–20.3)	27.7	11.9	0.11
2017	25 947	20.3 (19.2–21.4)	30.6	12.3	0.10
2018	26 809	21.0 (19.8–22.2)	30.6	9.2	0.11
2019	28 781	22.6 (21.3–23.8)	33.1	13.5	0.11
BS‐JSES					
2014	–	0.20 (0.16–0.24)	0.52	0	0.38
2015	–	0.25 (0.21–0.30)	0.65	0	0.36
2016	–	0.31 (0.26–0.36)	0.73	0	0.31
2017	–	0.36 (0.31–0.42)	0.75	0	0.30
2018	–	0.41 (0.36–0.47)	0.76	0.07	0.26
2019	–	0.48 (0.42–0.53)	0.90	0.07	0.24

*Note*: The incidence/number is reported per 100 000 person‐years. Gini coefficient varies between 0 (completely equity) and 1 (complete inequity) according to the degree of variation in the incidence/number among 47 prefectures.

Abbreviations: BS‐JSES, board‐certified surgeons by the Japan Society for Endoscopic Surgery; CI, confidence interval; RR, rectal resection.

### 3.4 Comparison of differences in the incidence/number of RC, RR, BS‐JSES, and the aging rate between urban and rural prefectures

Table 2 shows a comparison of differences in the incidence/number of RC, RR, BS‐JSES, and the aging rate between urban and rural prefectures. In particular, the incidence of RC, as well as the aging rate, was significantly higher in rural prefectures than in urban prefectures, while the number of BS‐JSES was significantly higher in urban prefectures than in rural prefectures. No significant differences were observed in the number of all RR, RR (laparoscopic), and RR (open) between the urban and rural prefectures. Furthermore, regarding all RR during 2014–2015 and RR (laparoscopic) during 2014–2019, the 95% confidence intervals for rural prefectures were included within those for urban prefectures, suggesting potential data equivalence.

**TABLE 2:** Urban/rural regional analysis for the incidence/number of rectal cancers, rectal resections, board‐certified surgeons by the Japan Society for Endoscopic Surgery, and the aging rate.

	Urban prefectures (*n* = 7)	Rural prefectures (*n* = 40)	*p* Value
	Mean (95% CI)	Mean (95% CI)	
**Rectal cancer per 100 000 person‐years**			
2016	40.1 (38.6–41.6)	43.9 (42.1–45.6)	**<0.01**
2017	38.8 (37.0–40.7)	42.1 (40.6–43.6)	**<0.05**
2018	38.8 (36.8–40.7)	42.3 (40.6–44.0)	**<0.05**
2019	39.8 (37.9–41.6)	43.4 (41.6–45.2)	**<0.05**
**All RR per 100 000 person‐years**			
2014	27.0 (24.7–29.3)	27.8 (26.7–28.8)	0.6
2015	29.4 (26.7–32.1)	30.3 (29.1–31.4)	0.6
2016	30.2 (27.6–32.7)	31.5 (30.1–32.8)	0.4
2017	29.8 (27.3–32.3)	31.2 (29.8–32.6)	0.4
2018	29.0 (26.9–31.0)	30.5 (29.2–31.8)	0.2
2019	29.7 (27.0–32.3)	31.1 (29.7–32.5)	0.4
**RR (open) per 100 000 person‐years**			
2014	11.2 (10.1–12.3)	12.3 (11.0–13.5)	0.2
2015	10.5 (9.3–11.7)	11.8 (10.6–13.0)	0.2
2016	10.8 (9.4–12.3)	12.3 (11.0–13.5)	0.2
2017	9.5 (8.0–11.1)	10.9 (9.7–12.1)	0.2
2018	8.2 (6.9–9.6)	9.5 (8.4–10.7)	0.2
2019	7.2 (5.7–8.7)	8.4 (7.3–9.6)	0.2
**RR (laparoscopic) per 100 000 person‐years**			
2014	15.8 (13.5–18.2)	15.5 (14.5–16.5)	0.8
2015	18.9 (15.7–22.2)	18.5 (17.2–19.8)	0.8
2016	19.4 (16.2–22.5)	19.2 (18.0–20.4)	0.9
2017	20.3 (17.3–23.3)	20.3 (19.1–21.5)	1.0
2018	20.8 (17.9–23.6)	21.0 (19.7–22.3)	0.9
2019	22.4 (19.2–25.7)	22.6 (21.2–24.0)	0.9
**BS‐JSES per 100 000 person‐years**			
2014	0.3 (0.2–0.4)	0.2 (0.1–0.2)	**<0.05**
2015	0.3 (0.2–0.4)	0.2 (0.2–0.3)	**<0.05**
2016	0.4 (0.3–0.5)	0.3 (0.2–0.4)	0.053
2017	0.5 (0.3–0.6)	0.3 (0.3–0.4)	**<0.05**
2018	0.5 (0.4–0.6)	0.4 (0.3–0.5)	**<0.05**
2019	0.6 (0.4–0.7)	0.4 (0.4–0.5)	**<0.05**
**Aging rate**			
2014	0.241 (0.232–0.251)	0.281 (0.274–0.289)	**<0.001**
2015	0.247 (0.238–0.257)	0.289 (0.282–0.297)	**<0.001**
2016	0.253 (0.242–0.264)	0.296 (0.289–0.304)	**<0.001**
2017	0.257 (0.245–0.269)	0.303 (0.295–0.311)	**<0.001**
2018	0.260 (0.247–0.273)	0.308 (0.300–0.316)	**<0.001**
2019	0.262 (0.249–0.276)	0.313 (0.305–0.321)	**<0.001**

*Note*: The results are expressed as the average (95% confidence interval). The bold value indicates a statistically significant difference (*p* < 0.05).

Abbreviations: BS‐JSES, board‐certified surgeons by the Japan Society for Endoscopic Surgery; RR, rectal resection.

## 4 DISCUSSION

Utilizing publicly accessible receipt‐based databases in Japan, we investigated the current patterns of RC and RRs. Our study confirmed the trend towards increasing laparoscopic RRs, as previously reported^16^, ^17^, ^18^. Specifically, the percentage of the number of laparoscopic RR of all RR was elevated from 54.4% (2014) to 73.8% (2019) in our study, while the percentage of laparoscopic surgery for RC was elevated from 9.8% (2005) to 52.8% (2016) in the ACS‐NSQIP database,^16^ and from 64.4% (2015) to 84.3% (2019) in a Japanese questionnaire‐based survey (response rate: 46.6% (2015), 43.0% (2019)).^17^, ^18^

In the analysis of regional disparities among the 47 prefectures, RC, all RRs, and laparoscopic RRs exhibited minimal disparities with Gini coefficients less than or equal to 0.1. These small inter‐prefectural disparities suggest a provision of consistent and uniform diagnosis and treatment and emphasize the significant demand for surgical interventions for malignancy. However, the Gini coefficient for open RR has trended upwards, showing moderate inequality after 2018 (Table 1). Considering that the annual national number of open RR is decreasing year by year (Figure 2), this may be because the weight of bias is increasing. Further investigation and monitoring are warranted.

The incidence of RC has exhibited a higher prevalence in rural prefectures in comparison to urban regions. We hypothesize that this elevated incidence in rural areas may also be due to the aging demographic composition (Figure 1 and Table 2), which could influence disease prevalence patterns. According to our previous study on gastric cancer and gastrectomy,^28^ both the incidence of gastric cancer and the number of gastrectomies were higher in rural prefectures, where the aging rate was also increased. Interestingly, despite the higher incidence of RC in rural patients, there was no statistical difference in the occurrence of RRs between the rural and urban groups. This particular finding suggests that RR is less commonly performed in rural areas, potentially because rural patients are seeking specialized surgical care in urban hospitals,^29^, ^30^ where RRs, particularly complex ones, are more common. This unexpected finding was not observed in our previous study.^28^ Therefore, this study provides valuable insight into the geographic distribution of RC surgery and offers relevant information for policy formulation. Subsequent longitudinal studies, incorporating future data, will be essential to further validate these findings.

This study has some limitations. First, our study excludes endoscopic treatment, chemotherapy, or palliative treatment, and instead focuses on open and laparoscopic RR. Therefore, it does not provide insight into the comprehensive management of RC. Second, the laparoscopic surgery in this study encompasses a minimal number of robotic procedures, and discerning between the two was not possible. Third, we were unable to include data pertaining patient demographics and medical history, such as cancer staging. These factors may confound our findings on regional disparities. Fourth, we included all RRs in our study, but a small subset of these resections may be for noncancerous cases (e.g. gastrointestinal stromal tumor, neuroendocrine tumor, and ulcerative colitis) or non‐RCs (e.g. anal skin cancer, and invasive cancer of adjacent organs), potentially influencing the overall interpretation of the findings. Fifth, regional disparities in healthcare between urban and rural prefectures are likely due to a complex interplay of numerous factors, potentially including some not captured in the available database. Lastly, differences in medical and social systems across different countries poses a challenge in generalizing our findings to other nations. Further research is warranted to address these limitations and to follow up the controversial benefit of laparoscopic RR.

In conclusion, this is the first observational epidemiological study that uses a nationwide database to investigate inter‐prefectural and urban–rural regional disparities in RC and RRs. We found that the approach to RR in Japan has gradually shifted from open to laparoscopic between 2014 and 2019. Additionally, it appears that RC and laparoscopic and all RRs exhibited minimal inter‐prefectural disparities. However, discernible differences in the incidence of RC and BS‐JSES exist between urban and rural prefectures, despite the minimal disparities in RRs regardless of approach. This pattern suggests a potential migration of surgical services from rural to urban areas. This preliminary study is expected to contribute to a basic epidemiological database for RC and RRs.

## AUTHOR CONTRIBUTIONS

Masamitsu Kido and Tomohiro Arita: Conceptualization. Masamitsu Kido and Katsutoshi Shoda: Methodology. Masamitsu Kido: Formal analysis and investigation. Masamitsu Kido: Writing–original draft preparation. Katsutoshi Shoda, Tomohiro Arita, Hiroki Shimizu, Jun Kiuchi, Kenji Nanishi, and Luying Yan: Writing–review and editing. Eigo Otsuji: Supervision.

## FUNDING INFORMATION

This study did not receive any specific grant from funding agencies in the public, commercial, or nonprofit sectors.

## CONFLICT OF INTEREST STATEMENT

The authors declare no conflict of interest for this article.

## ETHICS STATEMENT

Approval of the research protocol by an Institutional Reviewer Board: This study did not require institutional board approval or informed consent because of its retrospective nature and the use of legally anonymized public data.

Informed Consent: N/A.

Registry and the Registration No. of the study/trial: N/A.

Animal Studies: N/A.
